# Measuring Adiposity in Patients: The Utility of Body Mass Index (BMI), Percent Body Fat, and Leptin

**DOI:** 10.1371/journal.pone.0033308

**Published:** 2012-04-02

**Authors:** Nirav R. Shah, Eric R. Braverman

**Affiliations:** 1 Department of Medicine, New York University School of Medicine, New York, New York, United States of America; 2 PATH Foundation NY, New York, New York, United States of America; 3 Department of Neurosurgery, Weill-Cornell Medical College, New York, New York, United States of America; Aga Khan University, Pakistan

## Abstract

**Background:**

Obesity is a serious disease that is associated with an increased risk of diabetes, hypertension, heart disease, stroke, and cancer, among other diseases. The United States Centers for Disease Control and Prevention (CDC) estimates a 20% obesity rate in the 50 states, with 12 states having rates of over 30%. Currently, the body mass index (BMI) is most commonly used to determine adiposity. However, BMI presents as an inaccurate obesity classification method that underestimates the epidemic and contributes to failed treatment. In this study, we examine the effectiveness of precise biomarkers and duel-energy x-ray absorptiometry (DXA) to help diagnose and treat obesity.

**Methodology/Principal Findings:**

A cross-sectional study of adults with BMI, DXA, fasting leptin and insulin results were measured from 1998–2009. Of the participants, 63% were females, 37% were males, 75% white, with a mean age = 51.4 (SD = 14.2). Mean BMI was 27.3 (SD = 5.9) and mean percent body fat was 31.3% (SD = 9.3). BMI characterized 26% of the subjects as obese, while DXA indicated that 64% of them were obese. 39% of the subjects were classified as non-obese by BMI, but were found to be obese by DXA. BMI misclassified 25% men and 48% women. Meanwhile, a strong relationship was demonstrated between increased leptin and increased body fat.

**Conclusions/Significance:**

Our results demonstrate the prevalence of false-negative BMIs, increased misclassifications in women of advancing age, and the reliability of gender-specific revised BMI cutoffs. BMI underestimates obesity prevalence, especially in women with high leptin levels (>30 ng/mL). Clinicians can use leptin-revised levels to enhance the accuracy of BMI estimates of percentage body fat when DXA is unavailable.

## Introduction

Global trends of increasing obesity threaten public health and contribute to the burden of disease as much as smoking does [1], [2]. Obesity is associated with increased risk of diabetes, hypertension, heart disease, stroke, cancer, dyslipidemia, liver and gallbladder disease, sleep apnea and respiratory problems, osteoarthritis, abnormal menses and infertility [3]. Adiposity in mid-life strongly relates to reduced probability of healthy long term survival in women [4]. Obesity has become a priority of national, state and local public health efforts and in the care of individual patients. Thus, clinical detection of obese individuals has commensurately reached critical importance.

With the increasing importance of obesity detection, it is useful to reevaluate how body fat is determined. For adults, the body mass index (BMI) is commonly used. Its popularity stems in part from its convenience, safety, and minimal cost, and its use is widespread, despite not being able to distinguish lean body mass from fat mass [5]. The United States Centers for Disease Control and Prevention (CDC) explain: “For adults, overweight and obesity ranges are determined by using weight and height to calculate a number called the “body mass index” (BMI). BMI is used because, for most people, it correlates with their amount of body fat” [6]. However, the BMI is actually an indirect surrogate measurement considered imprecise [7], [8].

Recent estimates from NHANES, a nationally representative health examination survey, project that approximately 34% of adult Americans are overweight (defined as a BMI between 25–30 kg/m^2^) and an additional 34% are obese (BMI >30 kg/m^2^) [9]. In contrast, the CDC estimates rates of obesity over 20% in all 50 states with estimated rates over 30% in 12 states [http://www.cdc.gov/obesity]. These estimates are fundamental to US policy addressing the epidemic of obesity and are central to designing interventions aimed at curbing its growth, yet they may be flawed because they are based on the BMI.

The outdated BMI formula [BMI = weight in pounds/(height in inches)^2^×703], developed nearly 200 years ago by Quetelet, is not a measurement of adiposity, but merely an imprecise mathematical estimate [7], [8], [10]–[14]. Defining obesity based on percent body fat, as with BMI, also has arbitrary cut-points. In 1995, the World Health Organization (WHO) defined obesity based on a percent body fat ≥25% for men and ≥35% for women [15], while the most recent 2009 guidelines from the American Society of Bariatric Physicians (ASBP), an American Medical Association (AMA) specialty board, used percent body fat ≥25% for men and ≥30% for women. The ASBP percent body fat guidelines identify individuals that are suitable candidates for treatment for obesity with anorectic agents. Most studies comparing BMI with more accurate measures of adiposity used cutoffs of body fat >25% for men and >30% for women [16].

BMI ignores several important factors affecting adiposity. Greater loss of muscle mass leading to sarcopenic obesity in women occurs increasingly with age. BMI does not acknowledge this factor, exacerbating misclassifications [17], [18]. Furthermore, men's BMI also does not consider the inverse relationship between muscular strength and mortality [19]. It fails to take into account that men lose less muscle with age than women.

Statistical models have been created to explain variance in leptin with relation to insulin, gender, and BMI, but lack a variable of direct adiposity measurement such as DXA [20]. A fully equipped duel-energy x-ray absorptiometry (DXA) provides simultaneous measurements of muscle, bone mass and body adiposity. The ASBP uses both BMI and DXA as criteria for interventions.

Studies comparing DXA-derived percent body fat rates of obesity to BMI have, to date, focused mainly on women [12], [21] or imputed data on percent body fat for a substantial proportion of subjects [14]. We sought to characterize the degree of misclassification of obesity based on BMI using percent body fat from DXA in a large, unselected population, and to use the more accurate DXA derived measure to identify the optimal cut-points for defining obesity using BMI. Reclassifying obesity cut points is worth considering, as there is a population of individuals with a normal BMI who nonetheless have increased adiposity as determined by more sensitive methods; these are the so-called ‘normal weight obese.’ These individuals may have increased risk for medical comorbidities such as hyperlipidemia, coronary artery disease, hypertension, and diabetes [7]. Furthermore, in the intermediate ranges, BMI is not a good discriminator of cardiovascular risk; use of adiposity measures rather than BMI may be a better predictor, but have recently failed [22]–[25]. Therefore, there is a need to reclassify the obesity epidemic, identify clinically useful biomarkers, and clarify what the medical and scientific communities are measuring with BMI.

Although DXA is a direct measurement of fat and a better measure of adiposity than BMI, it is not a disease correlate. The attempts to find disease correlates to explain disparities between BMI and direct fat measurements have included leptin, insulin, ghrelin, and adiponectin [26]. Leptin, a 16 kDa peptide secreted primarily by adipocytes, regulates the body's energy balance by acting as a negative feedback adiposity signal, decreasing food intake and increasing energy expenditure. In individuals with leptin insensitive receptors, neither transport nor action is possible, and leptin levels rise [27]. Increased leptin is associated with the inflammatory process and possibly the entire increased morbidity of obesity [28], [29]. Individuals with leptin insensitivity and high levels of leptin have parallel comorbidities to normal weight obesity such as chronic inflammation, type II diabetes, hypertension, and myocardial injury [http://www.asbp.org/siterun_data/about_asbp/position_statements/doc7270523281295654373.html]. Therefore, it was appropriate to investigate whether leptin levels could correct for the disparity between DXA and BMI and be used to create a more accurate measure of obesity.

## Materials and Methods

We conducted a retrospective chart review of 9,088 patients who had ≥1 outpatient visits at a multispecialty private practice group in Manhattan (1998–2009). Patients who received a DXA scan within 3 weeks of their initial visit and whose height and weight were documented at first visit were eligible for study and signed written informed consent forms. DXA evaluation is routine in this wellness-focused practice; 71% of all patients seen from 1998 to 2009 received a DXA scan. 18% of patients had a DXA on the same day as their initial visit. Paper charts of those eligible patients identified from the DXA log were retrieved and reviewed by trained research assistants for demographic, height, weight, and selected laboratory and co-morbidity records. Patients selected for inclusion were adults (age = ≥18) with height, weight, and percent body fat (from DXA) available for analysis. No exclusions based on co-morbidities or other criteria were made. All height and weight data were abstracted in duplicate by separate raters to ensure accuracy; discrepancies were resolved by a final chart review and consensus.

BMI was calculated as weight (kg) divided by height (m) squared. Sectional and total percent body fat were attained from the Discovery Wi model of a Hologic DXA machine calibrated daily, which uses multiple pencil beam detectors and dual energy X-ray fan-beam to fat, muscle, and bone. A whole body scan was administered on each patient. QDR System software version 12.5 was used to analyze scans and provide percent body fat readings. All reported measurements of BMI, DXA and blood work were taken within 3 weeks of each other. Fasting insulin and leptin levels were drawn between 9:30 am and 3:00 pm. Fasting insulin levels were analyzed and reported by BioReference Lab. Leptin was measured by ELISA by ARUP Labs.

Institutional Review Board (IRB) approval was sought from PATH Foundation NY IRB and obtained prior to beginning research, and all investigators and personnel involved were trained in responsible conduct of research and protection of human subjects' information.

The National Institute of Health (NIH) criteria for obesity based on BMI were used to classify patients as obese (BMI ≥30). ASBP guidelines for percent body fat classify men as obese when body fat ≥25% and women as obese when body fat ≥30% [30]. Percent body fat (obese versus non-obese) was compared to BMI (obese versus non-obese) to determine percent agreement and disagreement. This analysis was conducted for all patients, all males, males by age category, all females, and females by age category.

A Receiver Operating Curve (ROC) analysis was used to identify cut points for BMI to optimize the area under the ROC curve (AUC), specifically sensitivity and specificity, relative to percent body fat. We conducted multiple logistic regression analyses using percent body fat (obese versus non-obese by ASBP criteria) as the outcome variable. The AUC metric was used to evaluate the strength of associations and improvement in the model when additional variables were added. Initial modeling evaluated the strength of association between percent body fat and BMI.

The effects of sex and age were evaluated to determine if either modified the association between percent body fat and BMI. If effect modification was present, then the study population was stratified and separate models were evaluated for each stratum. After regression models were developed accounting for BMI, sex, and age, other patient characteristics were added to the model to determine if the characteristic was associated with percent body fat. Additional analyses were conducted to evaluate the relationship between percent body fat and BMI, sex, age, fasting insulin and leptin levels. For preliminary analyses, percent body fat was defined as obese using cut-points described above (i.e. ≥30% for females and ≥25% for males). The primary predictor variables were BMI (continuous; categorical: <30 versus 30+; or ordinal: underweight, normal, over, Class I obese, Class II obese, Class III obese), sex, and age (continuous).

Subsequent analyses were conducted to examine if leptin or insulin levels were related to percent body fat. Currently accepted body fat percentage cut-points for obesity are 25% for men and 30% for women. For the purposes of this study, we identified the following groups based on percent body fat: for men <14% (Very low), 14%–17·9% (Fit), 18%–24.9% (Overweight), 25%–34.9% (Obese), 35%–39.9% (Morbidly obese), ≥40% (Super obese); for women <15% (Very low), 15%–24.9% (Fit), 25%–29.9% (Overweight), 30%–39.9% (Obese), 40%–44.9% (Morbidly Obese), ≥45% (Super obese). All statistical tests were two-sided with an alpha level of 5%, and conducted using SAS version 9.2.

## Results

A total of 1,393 adult patients (from 9,088) had both BMI and DXA derived percent body fat available for comparison. The population consisted of 63% women and 37% men, 75% white, with a mean age of 51.4 (SD = 14.2) (see Table 1). Mean BMI was 27.3 (SD = 5.9) and mean percent body fat was 31.3% (SD = 9.3).

**Table 1:** Summary of study population.

Variable	Total	Men	Women	p-value
**N**	**1,393**	518	875	N/A
Weight at time of DXA (kg), mean (SD)	76.61 (18·0)	86.77 (16.83)	70.62 (16.06)	<·0001
Height (meter), mean (SD)	1.67 (0.1)	1.76 (0.1)	1.62 (0.1)	<·0001
**BMI (kg/m^2^)**, mean (SD)	27.3 (5·9)	28.1 (5·4)	26.9 (6·2)	0.0001
Non-obese (BMI<30)	1031 (74%)	381 (74%)	650 (74%)	0.76
Obese (BMI 30+)	362 (26%)	137 (26%)	225 (26%)	
**Total Percent Body Fat** *	31.3 (9·3)	24.3 (7·0)	35.4 (7·8)	<.0001
Non-obese	507 (36%)	280 (54%)	227 (26%)	<.0001
Obese	886 (64%)	238 (46%)	648 (74%)	
Age at DXA (years), mean (SD)	51.4 (14·2)	51.8 (15·0)	51.2 (13·7)	0.42
Race: White, N (%)	1039 (75%)	423 (82%)	616 (70%)	<.0001
Black, N (%)	228 (16%)	56 (11%)	172 (20%)	
Hispanic, N (%)	76 (5%)	23 (4%)	53 (6%)	
Other, N (%)	50 (4%)	16 (3%)	34 (4%)	
Marital status: Married, N (%)	731 (53%)	295 (58%)	436 (50%)	0.0004
Single, N (%)	376 (27%)	145 (28%)	231 (27%)	
Divorced, N (%)	190 (14%)	52 (10%)	138 (16%)	
Widowed, N (%)	79 (6%)	18 (4%)	61 (7%)	
Unknown, N (%)	N = 17	N = 8	N = 9	
Insurance: Private, N (%)	1028 (74%)	368 (71%)	660 (75%)	0.19
Medicare, N (%)	173 (12%)	71 (14%)	102 (12%)	
Medicaid, N (%)	6 (<1%)	1 (<1%)	5 (<1%)	
None, N (%)	186 (13%)	78 (15%)	108 (12%)	
Systolic Blood Pressure (mmHg), mean (SD)	125.9 (18·3)	129.5 (17.2)	123.7 (18.6)	<.0001
Diastolic Blood Pressure (mmHg), mean (SD)	77.5 (10.4)	79.4 (9.9)	76.3 (10.6)	<.0001
Pulse (beats per minute), mean (SD)	72.1 (12.5)	70.·9 (12·6)	72·8 (12·4)	0·0099
Use cigarettes, N (%)	138 (11%)	72 (15%)	66 (8%)	<·0001
Use alcohol, N (%)	573 (46%)	262 (57%)	311 (39%)	<·0001
Leptin level (ng/mL), mean (SD)	26·1 (22·6)	13·3 (12·3)	31·7 (23·8)	<·0001
Insulin level (mIU/ml), mean (SD)	11·6 (15·4)	13·1 (17·1)	10·6 (14·0)	0·030

* Men were classified as non-obese based on a percent body fat <25% and obese for ≥25%; women were classified as non-obese based on a percent body fat <30% and obese for ≥30% (n = 1,393).

Blood pressure unknown for nine men and ten women.

Pulse unknown for 19 men and 25 women.

Cigarette use unknown for 49 men and 76 women.

Alcohol use unknown for 57 men and 85 women.

Leptin level unknown for 332 men and 450 women.

Insulin level unknown for 204 men and 397 women.

Table 2 demonstrates the discordance seen between classifications of obesity based on BMI versus percent body fat. While there was agreement for 60% of the sample, 39% were misclassified as non-obese based on BMI, while meeting obesity criteria based on percent body fat. Only 1% was classified as obese based on BMI, but non-obese by percent body fat. A total of 48% of women were misclassified as non-obese by BMI, but were found to be obese by percent body fat. In sharp contrast, 25% of men were misclassified as obese by BMI, but were in fact non-obese by percent body fat (i.e. the muscular body morphology).

**Table 2:** Percent body fat and BMI for all patients.

	MenN = 518	WomenN = 875	TotalN = 1393
**Concordant**			
BMI non-obese, % body fat non-obese	265 (51%)	227 (26%)	492 (35%)
BMI obese, % body fat obese	122 (24%)	225 (26%)	347 (25%)
**Discordant**			
BMI non-obese, % body fat obese	116 (22%)	423 (48%)	539 (39%)
BMI obese, % body fat non-obese	15 (3%)	0 (0%)	15 (1%)

**Figure 1:**
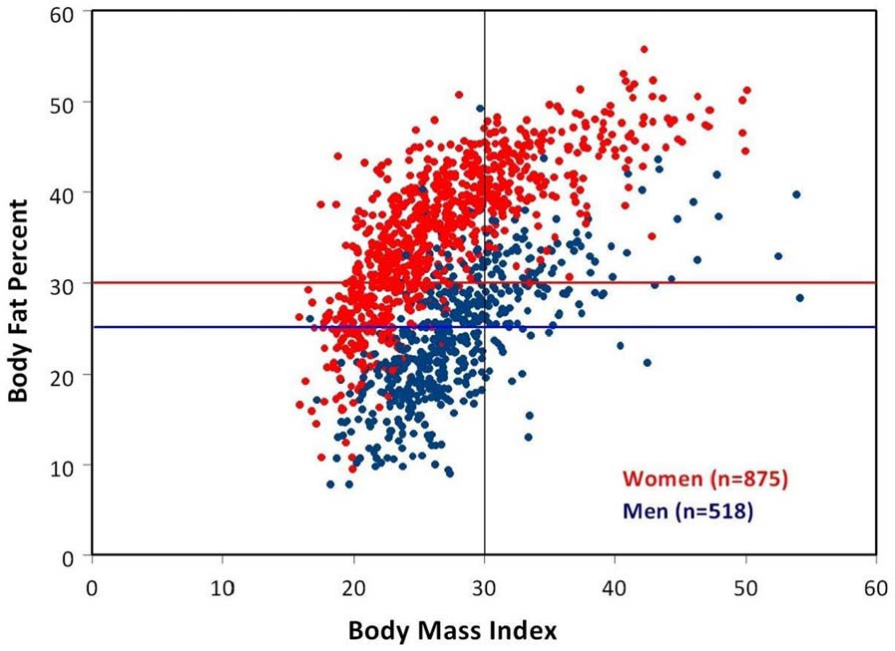
BMI versus Percent Body Fat in Scatter Plot. Women (red) who fall above red line are obese according to American Society of Bariatric Physicians criteria (DXA percent body fat: ≥30%). Men (blue) who fall above blue horizontal line are obese according to American Society of Bariatric Physicians criteria (DXA percent body fat: ≥25%). The upper left quadrant bordered by red horizontal line (body fat percent = 30%) and black vertical line (BMI = 30) demonstrates large number of women misclassified as “non-obese” by BMI yet “obese” by percent body fat.

**Figure 2:**
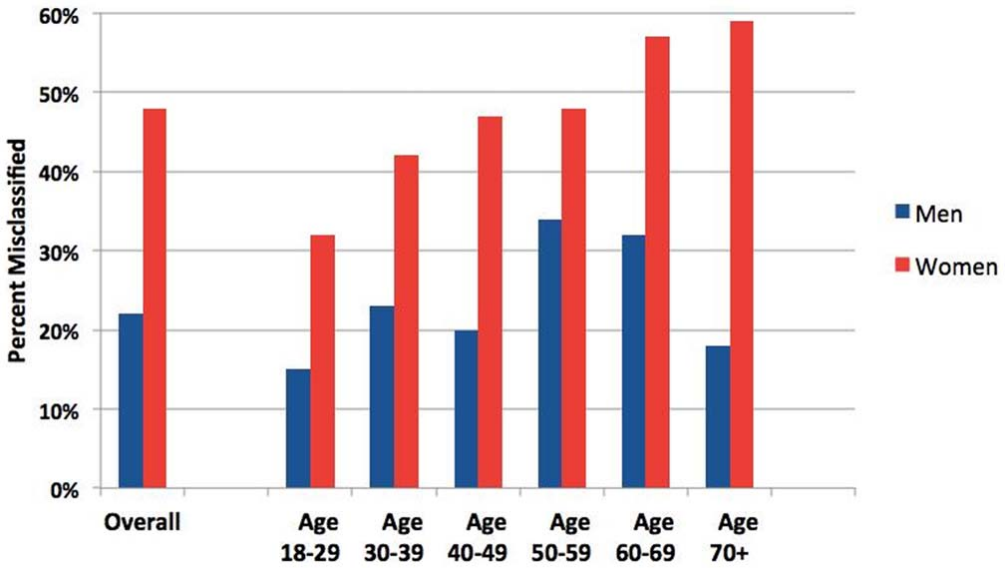
Percent Misclassified as Non-obese by BMI Statified by Age, and Sex (n = 539). Women demonstrate clear correlation between advancing age and increasing percent misclassification, with over half misclassified by age 60–69. This association is not apparent for men.

Figure 1 presents a scatter plot of BMI versus percent body fat. The upper left quadrant bordered by vertical BMI = 30% line and horizontal red line (women) or blue line (men), identifies the misclassified subjects who are non-obese based on BMI, but obese based on percent body fat. Examining these 39% (n = 539) of subjects in detail (see Figure 2), women had clear correlation between advancing age and % misclassification. 48% of women ages 50–59 misclassified, and 59% were misclassified by age 70+. This association with advancing age was not observed in men.

**Figure 3:**
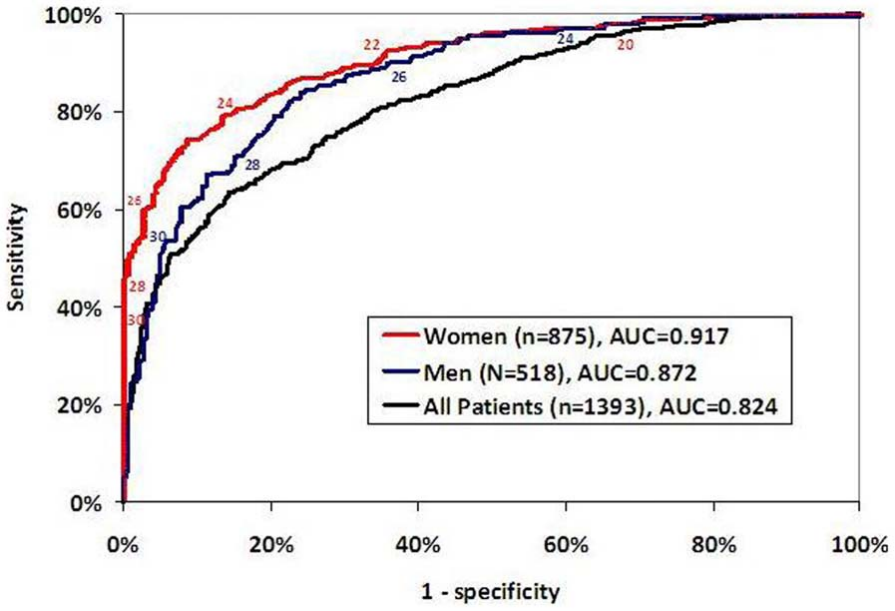
Receiver Operating Characteristic (ROC) Curve for Using BMI to Predict Obesity for Women. The area under the curve increases when stratified by sex. Numbers indicate the BMI cutoff value that corresponds to sensitivity/specificity along ROC curve. The BMI cutoff value that maximizes sensitivity and specificity is 24 for females (79% sensitivity and 87% specificity) and 28 for males (72% sensitivity and 83% specificity).

In regression modeling, BMI was a strong predictor of percent body fat whose association was modified by sex. Figure 3 contains the Receiver Operating Characteristic (ROC) curve for using BMI to predict obesity based on percent body fat. The area under the curve (AUC) was 0.824 for all patients, but was higher when stratified by sex (0.872 for males, 0.917 for females). For both models, age was a significant predictor of percent body fat, and AUC increased to 0.877 for males and 0.924 for females (ROC not shown). We attempted to identify new cut-points for BMI that would better categorize patients as obese, using percent body fat as the gold standard. Figure 3 shows that the BMI cutoff value that maximizes sensitivity and specificity is 24 for females (with 79% sensitivity and 87% specificity), and 28 for males (with 72% sensitivity and 83% specificity).

**Figure 4:**
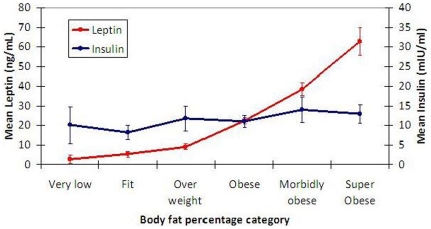
Comparison of Mean Leptin and Mean Insulin Across Percent Body Fat Categories. There is strong relationship between increased leptin and increased percent body fat, and no relationship between insulin and percent body fat. Error bars represent 95% confidence intervals for mean.

Figure 4 compares mean leptin and mean insulin across percent body fat categories. There is a strong relationship between increased leptin and increased percent body fat and the lack of relationship between insulin and percent body fat. Table 3 outlines the adjustment of the BMI score based on female leptin level and age to optimize the estimate of percent body fat, as defined by DXA. For example, a 45 year old woman with BMI of 23 and leptin level of 7 ng/mL (7 µg/L) has a percent body fat of approximately 23+5 = 28%. When BMI is >25, leptin levels do not add any new information to the equation, so we continue to add the average difference of 9 to adjust the BMI to better represent a woman's percent body fat. 13% of the total group (n = 89) fell into deficient or low normal leptin range (8.7% men, 4.4% women).

**Table 3:** BMI score adjustment based on female's leptin level and age to optimize the estimate of percent body fat.

BMI	Age	Leptin Level (ng/mL) (µg/L)	Adjustment to BMI to estimate % body fat
<25	18–40	0–4	0
		5–19	+5
		20+	+10
<25	41+	0–2	0
		3–9	+5
		10–19	+10
		20+	+15
>25	All	All	+9

Using new BMI cut-points for defining obesity would increase sensitivity with small tradeoffs in specificity. In women, BMI sensitivity to predict obesity (as defined by ≥30% body fat) increased from 35% at a BMI of 30 to 79% at BMI cutoff of 24, with specificity decreasing only 13% (100% to 87%). In men, BMI sensitivity increased from 51% with a BMI of 30 to 72% with a BMI of 28, with only a 12% loss of specificity (95% to 83%).

## Discussion

BMI significantly underestimates prevalence of obesity when compared to DXA direct measurement of percent body fat. Currently, no other blood test or biomarker has been correlated with the rate of obesity. The use of both DXA and leptin levels offers the opportunity for more precise characterization of adiposity and better management of obesity.

This misclassification was seen more commonly in women than in men and occurred more frequently with advancing age in women. A more appropriate cut-point for obesity with BMI is 24 for females and 28 for males (see Table 4). These new cut-points increased diagnostic sensitivity with small losses in specificity. Clinicians should consider using 24 as the BMI cut-point for obesity in women, in order to maximize diagnosis and prevention of obesity-related co-morbidities. Public health policymakers should also consider these more accurate cut-points in designing interventions. The Healthy People 2010 goal was to reduce rates of obesity (defined using BMI>30) from 23% in 1988–1994 to the target of 15%. Not only was this goal unmet, but in light of this data we may be much further behind than we thought. Our results document the scope of the problem of false-negative BMIs, emphasize the greater misclassification in women of advancing age, and confirm the improved precision available by gender specific revised cutoffs.

**Table 4:** Summary statistics for various BMI cut-off values predicting obesity as defined by percent body fat of >25% for men and >30% for women.

BMI cut-off value	Sensitivity	Specificity	PPV	NPV
**Men**				
**20**	100%	5%	47%	93%
**22**	100%	14%	50%	98%
**24**	98%	35%	56%	95%
**26**	90%	63%	67%	88%
**28**	72%	83%	79%	78%
**30**	51%	95%	89%	70%
**32**	34%	97%	90%	63%
**34**	23%	99%	95%	60%
**Women**				
**20**	99%	32%	80%	91%
**22**	92%	65%	88%	75%
**24**	79%	87%	95%	59%
**26**	62%	96%	98%	47%
**28**	48%	100%	100%	40%
**30**	35%	100%	100%	35%
**32**	23%	100%	100%	31%
**34**	17%	100%	100%	30%

PPV = positive predictive value; NPV = negative predictive value.

The use of leptin levels further improves precision of BMI adjustment, whereas insulin levels do not. With 91% of our patients with high leptin levels being women, our data confirm the greater effectiveness of BMI adjustment with leptin levels in women, attributable to a higher prevalence of hyperleptinemia among women. As significant lowering of leptin impacts long term weight control [31], [32], the idea of incorporating leptin adjustments into a more accurate diagnosis of obesity should be seriously considered. Further studies should be conducted for leptin measurements as a potentially useful tool in the management of obesity.

Greater loss of muscle mass (sarcopenic obesity) in women, with age, exacerbates the misclassifications of BMI [17], [18]. Women with increased adiposity with osteoporosis are at greater risk for impaired gait, disability, falls, and fractures [33]. In men, an inverse relationship has been shown between muscular strength and mortality which may be missed using BMI as a measure of adiposity [19]. A fully equipped DXA provides simultaneous measurements of muscle, bone mass and body adiposity. Since men lose less muscle with age than women, men's BMI should also take into account that men suffer from sarcopenia less than women. Models have been created to explain variance in leptin with relation to insulin, gender, and BMI, but have lacked a variable of direct adiposity measurement such as DXA [20]. Although this is new data, it appears likely that those who are older and all women will need a new classification of BMI – although our data are inclusive of all age groups. A definitive recommendation regarding which patients need DXA requires further study. The ASBP is using both BMI and DXA as criteria for interventions, and this may be a reasonable transition in public health policy. Some may prefer to use DXA alone, though the cost-effectiveness of this strategy is questionable. Given sufficient volume, DXA scans with body fat and bone density may be conducted efficiently at low cost.

Since a recent study showed that the significant lowering of leptin impacts long term weight control, the idea of utilizing leptin as a component in the national attack on obesity might be considered. To date, no other blood test or biomarker has correlated with the rate of obesity, while most of our other public health priorities have good biomarkers (e.g. A1c for diabetes, blood pressure for hypertension, etc.). Leptin measurements need further study as potentially useful in the management of obesity. While the strongest role for leptin is as a marker for improved outcomes, lowering elevated leptin has been associated with improved obesity and clinical outcomes [31], [32]. Numerous neurological, psychiatric, cardiac, and endocrine agents along with lifestyle changes have been associated with changing leptin and adiposity [31]. Inadvertently, a variety of medical disciplines may be choosing agents that cause weight gain for hyperleptinemic patients. The use of both DXA and leptin levels offer the opportunity for more precise characterization of adiposity and perhaps management of obesity. In the future, by measuring leptin, an entirely new range of treatment options may eventuate. Adiposity and hyperleptinemia are more significant than BMI in predicting high risk obesity. Measuring leptin may have value for BMI correction, predicting increased medical comorbidities related to hyperleptinemia and sarcopenia (including, but not limited to some cancers), and permanent weight loss [34]–[38].

### Limitations

Our data has several limitations. Our study was cross-sectional. Longitudinal data would allow quantification of outcomes related to adiposity, and future studies should evaluate the influence of adiposity on cardiometabolic and low bone mass density (BMD) outcomes, particularly in the “normal” BMI population. Although this study did not include longitudinal follow-up, it has already been established that increased adiposity correlates better than BMI with obesity co-morbidities [23], [24], [34], [35]. Furthermore, our subjects represented a convenience sample and had little racial/ethnic diversity. We were not able to accurately capture co-morbidities. We were also unable to compare other anthropometric indices, such as waist-to-hip ratio with corresponding DXA measurements, due to lack of hip circumference data. Previous research has suggested the utility of using lower cut-points for defining obesity. Romero-Corral [7] used a gold standard of percent body fat derived from bioelectrical impedance analysis to recommend a BMI>25.5 kg/m2 for women as an appropriate cut-point. In a population of postmenopausal sedentary women, Blew [21] recommended a cut-point of BMI>25, while Rahman [12] advocated for the use of race/ethnicity-specific BMI cut-points. NHANES [6] estimates that 28.6% of adult American women are overweight (BMI 25–30 kg/m2) and an additional 35.5% are obese (BMI>30 kg/m2). Shifting those currently considered overweight into the obese category would clarify the magnitude of the issue of obesity. By our cutoffs, 64.1% or about 99.8 million American women are obese.

BMI significantly underestimates adiposity. A better cutpoint for obesity with BMI is 24 for females and 28 for males. These body fat and leptin corrected BMI cutpoints are consistent with lower cutpoints for all-cause mortality in men and women [39]. Leptin levels enhance the precision of estimation in using BMI. The findings can be generalized since this was a cross-sectional study of the American population. Obesity, body fat and increased adiposity are more prevalent than the American public and American physicians are aware of. This is contributing greatly to multiple co-morbidities such as hyperlipidemia, coronary artery disease, hypertension, and diabetes. The current systematic underestimation of adiposity in large scale studies, and subsequent use of such studies for public health policy-making, can readily be corrected, resulting in a more appropriate sense of urgency and more cogent weighing of public health priorities. While BMI is less precise than direct adiposity measures in predicting medical co-morbidities, improving this globally used metric will have broad population health implications.

## References

[1] Jia H, Lubetkin EI (2010). Trends in quality-adjusted life-years lost contributed by smoking and obesity.. Am J Prev Med.

[2] Danaei G, Finucane MM, Lu Y, Singh GM, Cowan MJ (2011). National, regional, and global trends in fasting plasma glucose and diabetes prevalence since 1980: Systematic analysis of health examination surveys and epidemiological studies with 370 country-years and 2·7 million participants.. Lancet.

[3] Malnick SD, Knobler H (2006). The medical complications of obesity.. QJM.

[4] Sun Q, Townsend MK, Okereke OI, Franco OH, Hu FB (2009). Adiposity and weight change in mid-life in relation to healthy survival after age 70 in women: prospective cohort study.. BMJ.

[5] Gallagher D, Visser M, Sepulveda D, Pierson RN, Harris T (1996). How useful is body mass index for comparison of body fatness across age, sex, and ethnic groups?. Am J Epidemiol.

[6] NHLBI (1998). NHLBI Obesity Education Initiative Expert Panel on the Identification, Evaluation, and Treatment of Overweight and Obesity in Adults..

[7] Romero-Corral A, Somers VK, Sierra-Johnson J, Thomas RJ, Collazo-Clavell ML (2008). Accuracy of body mass index in diagnosing obesity in the adult general population.. Int J Obes.

[8] Fleming TR, DeMets DL (1996). Surrogate end points in clinical trials: are we being misled?. Ann Intern Med.

[9] Flegal KM, Carroll MD, Ogden CL, Curtin LR (2010). Prevalence and trends in obesity among US adults, 1999–2008.. JAMA.

[10] Flegal KM (2010). Commentary: the quest for weight standards.. Int J Epidemiol.

[11] Flegal KM, Shepherd JA, Looker AC, Graubard BI, Borrud LG (2009). Comparisons of percentage body fat, body mass index, waist circumference, and waist-stature ratio in adults.. Am J Clin Nutr.

[12] Rahman M, Berenson AB (2010). Accuracy of current body mass index obesity classification for white, black, and Hispanic reproductive-age women.. Obstet Gynecol.

[13] Razak F, Anand SS, Shannon H, Vuksan V, Davis B (2007). Defining obesity cut points in a multiethnic population.. Circulation.

[14] Sun Q, van Dam RM, Spiegelman D, Heymsfield SB, Willett WC (2010). Comparison of Dual-Energy X-Ray Absorptiometric and Anthropometric Measures of Adiposity in Relation to Adiposity-Related Biologic Factors.. Am J Epidemiol.

[15] World Health Organization (1995). Physical status: the use and interpretation of anthropometry. Report of a WHO Expert Committee.. World Health Organ Tech Rep Ser.

[16] Okorodudu DO, Jumean MF, Montori VM, Romero-Corral A, Somers VK (2010). Diagnostic performance of body mass index to identify obesity as defined by body adiposity: a systematic review and meta-analysis.. Int J Obes.

[17] Stenholm S, Harris TB, Rantanen T, Visser M, Kritchevsky SB (2008). Sarcopenic obesity: definition, cause and consequences.. Curr Opin Clin Nutr Metab Care.

[18] Di Monaco M, Vallero F, Di Monaco R, Tappero R (2011). Prevalence of sarcopenia and its association with osteoporosis in 313 older women following a hip fracture.. Arch Gerontol Geriatr.

[19] Ruiz JR, Sui X, Lobelo F, Morrow JR, Jackson AW (2008). Association between muscular strength and mortality in men: prospective cohort study.. BMJ.

[20] Zimmet P, Hodge A, Nicolson M, Staten M, de Courten M (1996). Serum leptin concentration, obesity, and insulin resistance in Western Samoans: cross sectional study.. BMJ.

[21] Blew RM, Sardinha LB, Milliken LA, Teixeira PJ, Going SB (2002). Assessing the validity of body mass index standards in early postmenopausal women.. Obes Res.

[22] De Lorenzo A, Del Gobbo V, Premrov MG, Bigioni M, Galvano F (2007). Normal-weight obese syndrome: early inflammation?. Am J Clin Nutr.

[23] Romero-Corral A, Somers VK, Sierra-Johnson J, Korenfeld Y, Boarin S (2010). Normal weight obesity: a risk factor for cardiometabolic dysregulation and cardiovascular mortality.. Eur Heart J.

[24] Shea JL, King MT, Yi Y, Gulliver W, Sun G (2011). Body fat percentage is associated with cardiometabolic dysregulation in BMI-defined normal weight subjects.. Nutr Metab Cardiovasc Dis.

[25] The Emerging Risk Factors Collaboration (2011). Separate and combined assocaitions of body-mass index and abdominal adiposity with cardiovascular disease: collaborative analysis of 58 prospective studies.. Lancet.

[26] Ursavas A, Ilcol YO, Nalci N, Karadag M, Ege E (2010). Ghrelin, leptin, adiponectin, and resistin levels in sleep apnea syndrome: Role of obesity.. Ann Thorac Med.

[27] Mantzoros CS (1999). The role of leptin in human obesity and disease: a review of current evidence.. Ann Intern Med.

[28] Chen K, Li F, Li J, Cai H, Strom S (2006). Induction of leptin resistence through direct interaction of C-reactive protein with leptin.. Nature Medicine.

[29] Martin SS, Qasim A, Reilly MP (2008). Leptin resistance: a possible interface of inflammation and metabolism in obesity-related cardiovascular disease.. J Am Coll Cardiol.

[30] American Society of Bariatric Physicians Website.. http://www.asbp.org/siterun_data/about_asbp/position_statements/doc7270523281295654373.html.

[31] Crujeiras AB, Goyenechea E, Abete I, Lage M, Carreira MC (2010). Weight regain after a diet-induced loss is predicted by higher baseline leptin and lower ghrelin plasma levels.. J Clin Endocrinol Metab.

[32] Koh KK, Park SM, Quon MJ (2008). Leptin and cardiovascular disease: response to therapeutic interventions.. Circulation.

[33] Walsh MC, Hunter GR, Livingstone MB (2006). Sarcopenia in premenopausal and postmenopausal women with osteopenia, osteoporosis an normal bone mineral density.. Osteoporos Int.

[34] Gomez-Amrosi J, Silva C, Galofre JC, Escalada J, Santos S (2011). Body adiposity and type 2 diabetes: increased with a high body fat percentage even having a normal BMI.. Obes J.

[35] Labruna G, Pasanisi F, Nardelli C, Caso R, Vitale DF (2011). High leptin/adiponectin ratio and serum triglycerides are associated with an “at- risk” phenotype in young severely obese patients.. Obes J.

[36] Prado CM, Lieffers JR, McCargar LJ, Reiman T, Sawyer MB (2008). Prevalence and clinical implications of sarcopenic obesity in patients with solid tumours of the respiratory and gastrointestinal tracts: a population-based study.. Lancet Oncol.

[37] Labayen I, Ortega FB, Ruiz JR, Lasa A, Simon E (2011). Role of baseline leptin and ghrelin levels on body weight and fat mass changes after an energy-restricted diet intervention in obese women: effects on energy metabolism.. J Clin Endicrinol Metab.

[38] Kelesidis T, Kelesidis I, Chou S, Mantzoros CS (2010). Narrative review: the role of leptin in human physiology: emerging clinical applications.. Ann Intern Med.

[39] Berrington de Gonzalez A, Hartage P, Cerhan JR, Flint AJ, Hannan L (2010). Body-mass index and mortality among 1.46 million white adults.. N Engl J Med.

